# Cost-effectiveness and cost-utility of hypertension and hyperlipidemia collaborative management between pharmacies and primary care in portugal alongside a trial compared with usual care (USFarmácia^®^)

**DOI:** 10.3389/fphar.2022.903270

**Published:** 2022-09-08

**Authors:** Suzete Costa, José Guerreiro, Inês Teixeira, Dennis K. Helling, João Pereira, Céu Mateus

**Affiliations:** ^1^ Escola Nacional de Saúde Pública (ENSP), Universidade NOVA de Lisboa, Lisboa, Portugal; ^2^ Institute for Evidence-Based Health (ISBE), Lisboa, Portugal; ^3^ Centre for Health Evaluation and Research (CEFAR), Infosaúde, Associação Nacional das Farmácias (ANF), Lisboa, Portugal; ^4^ Skaggs School of Pharmacy and Pharmaceutical Sciences, University of Colorado, Denver, Colorado, United States; ^5^ Centro de Investigação em Saúde Pública (CISP), and Comprehensive Health Research Centre (CHRC), Lisboa, Portugal; ^6^ Health Economics at Lancaster, Division of Health Research, Lancaster University, Lancaster, United Kingdom

**Keywords:** community pharmacy, primary care, hypertension, hyperlipidemia, cost-effectiveness, cost-utility, economic, real-world

## Abstract

**Background:** There is little experience in the economic evaluation of pharmacy/primary care collaborative health interventions using interprofessional technology-driven communication under real-world conditions. This study aimed to conduct cost-effectiveness and cost-utility analyses of a collaborative care intervention in hypertension and hyperlipidemia management between pharmacies and primary care versus usual (fragmented) care alongside a trial.

**Methods:** An economic evaluation was conducted alongside a 6-month pragmatic quasi-experimental controlled trial. Data sources included primary care clinical software; pharmacy dispensing software; patient telephone surveys; and published literature. The target population was adult patients on hypertension and/or lipid-lowering medication. The perspective was societal. We collected patient-level data on resource use to estimate trial costs. Effect outcomes included blood pressure (BP) and quality-adjusted life years (QALYs). Bootstrapping was used to estimate uncertainty around the incremental cost-effectiveness and cost-utility ratios. Cost-effectiveness planes and acceptability curves were estimated.

**Results:** The intervention was not shown to have reasonable levels of cost-effectiveness or cost-utility when compared to usual care as denoted by the levels of uncertainty expressed in wide confidence intervals. The probability of the intervention being cost-effective is 28% at the threshold of €20,000 per QALY gained and 57% at the threshold of €500 per mmHg systolic BP decrease.

**Conclusion:** Considering the limitations of the trial which affected effectiveness and economic outcomes, our results are not generalizable for community pharmacy and primary care in Portugal. This research offers, however, valuable lessons on methods and strategies that can be used in future economic evaluations of collaborative public health interventions with the potential for reimbursement.

**Clinical trial registration:** https://www.isrctn.com/ISRCTN13410498, identifier ISRCTN13410498

## 1 Introduction

In 2013, 12.3% of all hospital admissions in Portugal were due to Ambulatory Care Sensitive Conditions (ACSCs), and 93.7% occurred following emergency room (ER) visits. The third and fourth most common ACSCs were heart failure and hypertensive heart disease, often as a consequence of poorly managed hypertension and hyperlipidemia, which creates an opportunity to improve service delivery (World Health Organization Regional Office for Europe, 2016).

We found two overviews of systematic reviews covering a wide variety of pharmacist-provided interventions including, but not restricted to, hypertension and hyperlipidemia. Mossialos et al. (2013) looked at 33 systematic reviews and established positive trends in the effectiveness of pharmacist-provided disease management (including cardiovascular risk, hypertension, lipid management, and adherence to antihypertensive medication), screening, and referral, and smoking cessation. Of these, six addressed hypertension and/or hyperlipidemia management provided by pharmacists, of which five presented positive findings in blood pressure and lipid parameters, and one presented positive findings in systolic blood pressure but mixed findings in adherence and quality-of-life. Most of the interventions were also complex and multifaceted, while some systematic reviews may not be exclusive to community pharmacists; overall, Mossialos et al. (2013) consider that “positive evidence was found supporting community pharmacist interventions for… hyperlipidemia,… blood pressure control, and adherence to antihypertensive therapy”. The overview of Rotta et al. (2015) included 49 systematic reviews and also determined positive trends in the effectiveness of disease management, including hypertension, provided by pharmacists. Of these, six addressed hypertension management, and all of them showed positive results in blood pressure reduction. The authors also report that these interventions are multifaceted. The overview identified two systematic reviews focusing on hyperlipidemia management by pharmacists. Both reviews showed positive findings in total cholesterol improvement, but the authors state that one review could not establish improvements in other lipid parameters. However, when looking at this review of 23 studies, only eight were in the community pharmacy setting, and all of them demonstrated positive findings in all lipid parameters in the community pharmacy setting.

A meta-analysis of 39 randomized controlled trials comprising 14,224 patients focusing on pharmacist intervention in hypertension established the impact of these interventions in improving systolic blood pressure (BP) by -7.6 mmHg (95% CI, from −9.0 to −6.3 mmHg) and diastolic BP by −3.9 mmHg (95% CI, from −5.1 to −2.8 mmHg) versus usual care (Santschi et al., 2014). Pharmacist interventions included patient education, feedback to physicians, and medication management. A quasi-experimental controlled trial conducted in Portuguese pharmacies in 1999, comprising 109 patients, reached similar results of −7 mmHg in systolic BP and −2 mmHg in diastolic BP (Costa, 2001). A systematic review of 21 randomized controlled trials comprising 5,416 patients established the impact of pharmacist interventions in improving lipid parameters versus usual care. A subset of 10 studies of this review using 1,196 patients established improvements in total cholesterol (TC) of −15.2 mg/dl (95% CI, from -24 to 6.4 mg/dl) (Charrois et al., 2012).

In the context of limited and scarce resources, it may be important to invest in health technologies not product-related (public health interventions) that make use of the walk-in access of pharmacies, equitable geographical distribution, high frequency of patient interactions, patient trust, long opening hours, and highly skilled professionals in pharmacies, provided they contribute to optimize the benefits of medicines (compliance, safety, and effectiveness) and improve health outcomes at acceptable costs.

The economic evaluation of public health interventions in collaborative care environments may contribute to informing decisions in reimbursing such services and their successful expansion, continuation, or justification (Schumock and Butler, 2003).

We performed a comprehensive search in the following databases throughout September 2018 (updated January 2020): MEDLINE and EMBASE *via* the OVID SP interface, Web of Science, Database of Abstracts of Reviews of Effects (DARE), NHS Economic Evaluation Database (NHS EED) and HTA *via* the CRD database, and Tufts CEA Registry to identify economic evaluations of pharmacy-based hypertension or hyperlipidemia management according to pre-defined inclusion criteria.

One researcher performed the screening of titles, abstracts, and full-text articles. Included and excluded full-text articles were reviewed for eligibility by another researcher. Data were extracted by one researcher and used by another researcher. We present a summary of the results for context.

We found 11 studies on the economic evaluation of pharmacy-based hypertension management of which three are partial evaluations reporting costs and outcomes (Bond et al., 2007; Bunting et al., 2008; Wertz et al., 2012), four are cost-utility analyses (CUA) (Elliott et al., 2017; Marra et al., 2017; Bosmans et al., 2019; Twigg et al., 2019), two are cost-benefit analyses (CBA) (Côté et al., 2003; Patterson et al., 2016), one is a cost-effectiveness analysis (CEA) (Shireman and Svarstad, 2016), and one includes willingness-to-pay using discrete choice but is not a full CBA (Fletcher et al., 2019). Four of these studies were conducted in the United States, four in the United Kingdom, two in Canada, and one in the Netherlands.

Shireman and Svarstad (2016) estimated an incremental cost-effectiveness ratio (ICER) of $22.2 (US dollars) per mmHg systolic BP improvement and $66.0 per mmHg diastolic BP improvement. Elliott et al. (2017) established cost-utility for a pharmacy-based adherence intervention in hypertension, and the incremental cost-utility ratio (ICUR) was estimated to be -£115.5 (Sterling pounds) per quality-adjusted life year (QALY) (dominant, less costly, and more effective) with a 93% probability of being cost-effective at a £20,000 threshold value. Marra et al. (2017) estimated an ICUR of −$21,216.67 (Canadian dollars) per QALY (dominant). Twigg et al. (2019) estimated an ICUR of £8,495.29 (Sterling pounds) per QALY with a 97% probability of being cost-effective at a £20,000 threshold value. Bosmans et al. (2019) estimated an ICUR of €59,979 per QALY with a 36% probability of being cost-effective at a €20,000 threshold value. Both Elliott and Marra’s studies were able to demonstrate that pharmacy-based interventions in hypertension management were less costly and more effective than usual care; while this may be unusual in cost-utility studies of medicines, a pivotal study provided a powerful message that many public health interventions are indeed cost-effective, well below the NICE threshold of £20,000–30,000 per QALY. This study looked at 200 NICE public health interventions and found that 15% of public health interventions were cost-saving, and 70.5% were cost-effective at the threshold of £20,000 per QALY, of which 49% are cost-effective at the threshold of just £1,000 per QALY (Owen et al., 2012). Of all the seven CEA and CUA studies in pharmacy-based hypertension and hyperlipidemia management, only one, Bosmans et al. (2019), shows the intervention in hypertension management that does not appear to be cost-effective at the usual threshold. One possible explanation could be that this study looked at self-reported adherence, as an effect measure, instead of blood pressure.

In addition, we found four studies on the economic evaluation of pharmacy-based hyperlipidemia management of which two are partial evaluations reporting costs and outcomes (Ditusa et al., 2001; Simpson et al., 2001) and two are CUA (van Boven et al., 2014; Vegter et al., 2014). These last two studies address the same intervention and estimate an ICUR of approx. €5,000 per QALY.

Planning and conducting a well-designed trial for the assessment of effectiveness and other dimensions should precede the economic evaluation of pharmacy-based public health interventions (Costa et al., 2019). Therefore, we first developed a quasi-experimental pragmatic controlled trial to assess the effectiveness of the first collaborative care intervention between pharmacies and primary care in hypertension and hyperlipidemia management in Portugal (USFarmácia^®^).

Despite non-significant results for effectiveness, we used the main findings in BP outcome to experiment a proof-of-concept cost-effectiveness study. Our goal was to conduct an economic evaluation of an intervention in a real-world context using primary data from various data sources—regardless of the results of the blood pressure or quality-of-life change—and learn from that first experiment to improve future trial-based economic evaluation studies. We used BP incremental effectiveness per month and determined the overall BP change for the 6-month trial period for cost-effectiveness.

This study presents the second part of this research project, addressing the economic evaluation of the collaborative pharmacy-based public health intervention with primary care.

The main research question of this study was to carry out cost-effectiveness and cost-utility analyses of a real-world collaborative intervention in hypertension and/or hyperlipidemia using information technology (IT) between pharmacies and a National Health Service (NHS) primary care family health unit (“Unidade de Saúde Familiar,” or USF) versus usual (fragmented) care to guide and improve future experiments with the potential of reimbursement.

## 2 Methods

This is a trial-based economic evaluation.

We followed the proposed methodological approach for planning, conducting, and reporting an economic evaluation of pharmacy-based public health interventions (Costa et al., 2019): we used the updated Consolidated Health Economic Evaluation Reporting Standards (CHEERS) 2022 checklist (Husereau et al., 2022), assisted by recommendations for economic evaluations of public health interventions (National Institute for Health and Clinical Excellence, 2012) and economic evaluations alongside trials (Ramsey et al., 2005; Ramsey et al., 2015).

### 2.1 Population, setting, and location

This was a multicenter, pragmatic, quasi-experimental controlled trial of a collaborative care intervention between community pharmacies and a primary care USF in Portugal for the management of adult patients on hypertensive and/or lipid-lowering medication versus usual care involving seven interventions and 13 control pharmacies.

Demographics, socioeconomic, and clinical variables of the study population are reported in the Results section.

### 2.2 Intervention and comparator

Methods for the USFarmácia^®^ trial were described in the trial register [ISRCTN13410498]. Trial design, challenges, and effectiveness results are reported in a separate article submitted for publication at the time of writing this manuscript, but we briefly report key methods and findings to provide some context.

In short, patients were recruited in community pharmacies according to inclusion criteria: adult patients of selected USFs; on medication for hypertension and/or hyperlipidemia; and holders of an NHS number. We excluded diabetic patients.

The study intervention consisted of hypertension and/or hyperlipidemia management within a collaborative care framework between intervention pharmacies and primary care according to pre-defined integrated care pathways (ICPs). ICPs consist of consensus-based clinical decision algorithms integrated into the pharmacy dispensing software with data exchange between pharmacies and primary care. The planned IT-driven components of the collaborative health intervention package included 1) point-of-care measurements; 2) cardiovascular risk assessment; 3) medication management; 4) request for repeat prescription; 5) lifestyle counseling; 6) referral and direct request for a medical appointment as per ICP; 7) feedback from USF and follow-up at the pharmacy as per ICP; 8) refill text reminder to the patient; 9) interprofessional meetings between pharmacists, physicians, and nurses (quality circles); (10) reporting a potential adverse drug event (ADE). Intervention pharmacies were reimbursed for a limited number of pharmacy visits and point-of-care measurements per patient to mimic a real-world routine intervention.

The intervention was operational between May 2018 and July 2019.

Some of these components were implemented for the first time in Portugal using this project: 1) decision algorithms integrated into the pharmacy dispensing software; 2) data exchange between pharmacies and primary care on point-of-care measurements, cardiovascular risk assessment, medicines dispensed, request for a repeat prescription when the last package dispensed, and direct request for medical appointment; 3) refill reminder sent from pharmacy software to patient cell phone 10 days before the end of package; 4) quality circles; 5) experimental bundled reimbursement to intervention pharmacies.

The study’s comparator was usual care (control group) provided by pharmacies and primary care as it reflects the current practice with the potential to improve. The components mentioned earlier were not present in the usual care provided in control primary care units and pharmacies.

### 2.3 Key findings (effectiveness study)

We used a controlled interrupted time series (CITS) to look at the trend effect in systolic blood pressure considering all interventions and control patients’ BP measurements recorded in the primary care database 6 months before the onset of intervention and 6 months after. The trend effect of the intervention vs. control group in systolic BP change per month although negative (−0.43 mmHg) is not significant (95% CI, −4.93 to 4.07). The same applies to diastolic BP change per month (0.48 mmHg; 95% CI −2.00–2.96).

### 2.4 Perspective

The cost of implementing the intervention/usual care is derived from the limited societal perspective as per recommendations of economic evaluations alongside trials (Ramsey et al., 2005; Ramsey et al., 2015) and as per the ISPOR Drug Task Force Report II (Garrison et al., 2010). This includes costs to both the NHS, providers, and patients, including indirect costs.

### 2.5 Time horizon

This research study used panel data. We collected all patient-level costs and outcomes alongside the trial for 6 ± 2 months, the minimum time frame reported in the literature to produce changes in BP and/or TC.

### 2.6 Discount rate

We applied no discount rate due to a time horizon <1 year.

### 2.7 Selection and measurement of effectiveness outcomes

Blood pressure is a measure of disease control as defined in the International Consortium for Health Outcomes Measurement (ICHOM) Standard Set for hypertension in low- and middle-income countries (International Consortium for Health Outcomes Measurement, 2017). Systolic BP is a natural valid intermediate outcome since it is a predictor of the 10-year risk of fatal cardiovascular disease (SCORE) along with gender, age, and smoking status (Piepoli et al., 2016) and is routinely measured by pharmacists, physicians, and nurses.

We collected BP measured by primary care providers alongside the trial for CEA from primary care prescribing and clinical software. Sample size calculation was calculated for BP change in the effectiveness study.

### 2.8 Selection, measurement, and valuation of quality-of-life

We also collected quality-of-life outcomes to derive QALYs to experiment with a CUA. In line with recommendations (Ferreira et al., 2014), quality-of-life was measured using the EQ-5D-3L™ which uses five health dimensions (mobility, self-care, usual activities, pain/discomfort, and anxiety/depression) rated on a 3-level severity scale (no problems, some problems, and extreme problems) (Ferreira et al., 2013) at baseline and 6 months through a patient telephone survey after seeking for appropriate license agreement.

We used the EQ-5D-3L™ value set estimated from a representative sample of the Portuguese population (Ferreira et al., 2014) to convert patients’ responses to the EQ-5D-3L™ telephone questionnaire at baseline and 6 months into single utility levels (a scale where zero is equal to death and one is full health). In addition, a visual analog scale (VAS) was used to measure patients’ health status with scores that ranged from 0 (worst) to 100 (best) health state.

### 2.9 Measurement and valuation of resources and costs

We estimated costs from the USFarmácia^®^ trial. We collected real-world longitudinal patient-level prospective data on the quality of life, use of health service resources, time and travel costs, and days lost.

Intervention start-up costs (e.g., training and trial monitoring) are excluded so that interventions are evaluated and compared as if they were operating under steady-state conditions when expanding an established intervention, as would be the case in the roll-out of a reimbursed intervention (Foster et al., 2003).

We used the three sources for data collection (Box 1).

**BOX 1:** Resource use data and sources for quantities.


Items	Time point recorded	Data source (quantities)
Pharmacy visits Pharmacy point-of-care measurements and tests	All available data points 6 ± 2 M after patient enrolment (No intervention prior to enrolment)	Pharmacy dispensing software
GP visits Nurse visits USF point-of-care measurements and tests	All available data points 6 ± 2 M prior to patient enrolment 6 ± 2 M after patient enrolment	Primary care prescribing and clinical software
Prescribed anti-hypertensive/lipid-lowering medication	All available data points 6 ± 2 M prior to patient enrolment 6 ± 2 M after patient enrolment	Primary care prescribing and clinical software
Quality of life	0 and 6 months	Patient telephone survey using EuroQol-5 dimension-3 level instrument (EQ-5D-3L) and Visual Analog Scale (EQ-VAS)
Primary care + hospital ER visits Hospital outpatient visits Days in hospital Working days lost Travel + waiting time to USF/Pharmacy Means of transport + km or cost	0 and 6 months (in the previous 6 months)	Patient telephone survey

GP, general practitioner; USF, primary care family health unit; ER, emergency room.

These resources were combined with available costs to derive patient-level total costs based on general recommended methods (Drummond et al., 2005), recommendations for cost estimation and valuation in Portugal (Mateus, 2009) including published NHS unit costs, and micro-costing including time-driven activity-based costing (TDABC) to estimate the cost of pharmacy and primary care interventions (Elliott et al., 2008; Sach et al., 2015; Twigg et al., 2019; Cidav et al., 2020) as this is an accurate method to capture real costs in healthcare and drive value-based health care (Keel et al., 2017; Etges et al., 2020).

We used NHS data, including diagnostic-related groups (“Grupos de Diagnóstico Homogéneo,” or GDH) for published unit costs of health care resources, point-of-care measurements, and diagnostic tests (Portaria, 2017).

We assumed the hospital admission cost of the most frequent cause of hospital admission in hypertension/hyperlipidemia—ischemic stroke GDH code 45—and severity level 1.

In the absence of published general practitioner (GP) visit unit cost, we used the estimate of a GP visit unit cost in 2011 in Portugal (excluding medication and diagnostic tests) performed by other authors in a research study and adjusted to 2018 by applying the Portuguese Consumer Price Index (Gouveia et al., 2012). The resulting cost is similar to the hospital outpatient visit unit cost published in the 2017 NHS tariff after adjustment for 2018 as well.

We used a pharmacy cost of €0.75 per minute provided by the Centre of Health Evaluation and Research (CEFAR) of the National Association of Pharmacies (‘Associação Nacional das Farmácias or ANF) which was determined based on operating costs in 2016 (Antão and Grenha, 2018), average operating hours per week in 2018 (Farmácias Portuguesas, 2018), and average minimum pharmacist salary in 2018 (dre, 2018) for Portuguese pharmacies. Operating costs used are also similar to estimates in 2016 by the Portuguese Central Bank (“Banco de Portugal”). Average operating hours per week used were also provided by each pharmacy to the Portuguese Medicines and Health Technology Agency Infarmed in March 2018.

This pharmacy cost was based on conservative assumptions and was used by ANF Pharmacy Customer Loyalty Program Saúda^®^ to reimburse intervention pharmacies for a limited number of pharmacy visits per patient as per ICP. We considered 30 min for the first pharmacy visit and 15 min for follow-up visits based on actual time reported by pharmacists for similar patient care pharmacy visits in a pharmacy daily survey used in a previous study (Gouveia et al., 2009). This cost does not include the profit margin for the pharmacy; hence, it does not equal a reimbursed fee rate, and it was left out of the cost estimate. This cost per minute is also very similar to €0.74 or €0.78 per minute, which we can infer from another Portuguese research study when dividing the average cost of each activity by the average time spent (Gregório et al., 2016).

We considered the following Anatomical Therapeutic Chemical (ATC) for antihypertensive and lipid-lowering medications: C02 (antihypertensives); C03 (diuretics); C07 (beta blockers); C08 (calcium channel blockers); C09 (agents acting on the renin–angiotensin system); and C10 (lipid-modifying agents). We used the retail price for medicines prescribed by the national product code (“Código Nacional de Produto” or CNP) and the average retail price of the national code for electronic prescribing (“Código Nacional para a Prescrição Electrónica de Medicamentos,” or CNPEM) for medicines prescribed by international nonproprietary name (INN), pharmaceutical form, dosage, and pack size. We used 3rd quarter 2018 retail prices.

We used the human capital approach for paid and unpaid productivity loss costs, considering the societal perspective (Hubens et al., 2021). This included the cost of work loss attributable to absenteeism in workers and unpaid work of retired, disabled, unemployed, or performing household tasks who are able to perform activities replaceable by a third hired person, for example, household activities, informal care to more dependent people or grandchildren, volunteer work, or paid tasks outside the formal workforce (Cylus et al., 2019), since the average age of our study population is just slightly below retirement age 66.3 in 2018.

We used the formula from Mitchell and Bates for productivity loss (Mitchell and Bates, 2011), modified by the authors to fit the Portuguese context. To derive the unit cost of work loss due to hypertension and/or hyperlipidemia, we calculated 1) mean hourly wage rate based on average monthly wages plus benefits in Portugal in 2018 (GEP/MSESS, MTSSS, PORDATA, 2018) multiplied by 14 payments per year and divided by the number of working days in 2018 (The Workingdays Team, 2018) multiplied by the daily fraction of average working hours per week in 2018 (Eurostat, INE, PORDATA, 2018); 2) mean daily compensation for full-time employees multiplied by an average wage “multiplier” of 1.55, defined as the cost to the health system in Portugal of a 30-day absence as a proportion (55%) of the absent worker’s daily wage (Segurança Social, 2018).

We used the same formula to estimate the daily cost of patient time lost in travel and waiting time in GP, nurse, and pharmacy visits, but without adding the average wage “multiplier,” as this was not a paid sick leave.

X¯hourly wage rate=X¯monthly wages 2018 × 14Nworking days 2018  (X¯working hours per week 20185), (1)

X¯daily cost work loss= X¯hourly wage rate (X¯working hours per week 20185) ×1.55. (2)

For transportation costs, we used direct costs reported by patients in the telephone survey. In the case of patients reporting km (car), we used published unit cost per km as per legislation (Portaria, 2008) in its updated version of 2010 (Decreto-Lei, 2010) and adjusted it to 2018 prices.

### 2.10 Currency, price date, and conversion

All costs were reported in euro (€), resource use data refer to 2018, and price date was reported for each unit cost. When unit costs or prices were available from previous years, we adjusted to 2018 (trial onset year) by applying the Portuguese Consumer Price Index (INE, 2018) to reflect the time period of patient enrolment.

### 2.11 Measurement of patient demographics, socioeconomic, and clinical data

In addition to health outcomes and cost data, we collected other patient data.

See Supplementary File S1 (Methods: Measurement of Patient Demographics, Socioeconomic, and Clinical Data).

### 2.12 Analytics and assumptions

Patient demographics and case-mix variables were summarized using descriptive statistics and were presented by the study arm. We compared the baseline characteristics between intervention and control patients, including EQ-5D-3L™ baseline score and cost data, to assess baseline equivalence. We compared EQ-5D-3L between groups using the test for comparison of the treatment group values and within groups using the Wilcoxon signed-rank test for pairwise comparisons baseline/6 months. Significance was set at *p* < 0.05.

We considered an intention-to-treat (ITT) population, including patients regardless of the degree of intervention exposure, as this was a pragmatic trial.

Handling of missing data in the primary analysis consisted of available-case analysis for missing cost data (Briggs et al., 2003), and completed-case analysis for missing EQ-5D-3L™, assuming missing at random (MAR). Since the MAR assumption had already been tested in the effectiveness study and no significant differences were found between patients with missing 6-month BP and/or TC levels and patients with completed 6-month assessment for their baseline characteristics, we assumed MAR would also hold valid for patients with missing 6-month quality-of-life versus patients with completed 6-month assessment.

We calculated mean costs per patient for each item of resource use for each group over the 6-month follow-up period by summing total costs for each item of resource use and dividing by the total number of patients in each item. We then aggregated mean costs per patient for each item of resource use to estimate the total mean cost per patient in each group. The mean cost difference between groups over the 6 month-trial provided an estimate of the incremental cost.

After converting patients’ responses to the EQ-5D-3L™ telephone questionnaire into single utility levels, QALY scores were calculated for the 6 months after baseline using the area under the curve (AUC) approach (Manca et al., 2005) multiplied by 0.5 (fraction of the year corresponding to 6 months), where baseline adjustment was undertaken using GLM to estimate the QALY gain over the 6 months. We estimated the QALY gain in each group for all patients who completed the EQ-5D-3L™ at baseline and 6 months.

In the base-case analysis for cost-effectiveness, we included patients if the incremental cost and incremental effectiveness could be calculated. We used the same approach for the base-case analysis for cost-utility.

We estimated the mean incremental cost and effectiveness/QALY gain per patient and subsequently used it to estimate both the ICER and the ICUR generated by collaborative care over usual care using the following equation:

$$\frac{Cost_{Intervention\;}-\;Cost_{Control}\;}{Effectiveness\;{\left(or\;QALY\right)}_{Intervention\;}-\;Effectiveness\;{\left(or\;QALY\right)}_{Control}\;}.$$

All statistical analyses were conducted using SAS enterprise guide v7.15.

### 2.13 Characterizing heterogeneity

We explored changes in EQ-5D-3L™ scores at baseline/6 months for the following patient subgroups: uncontrolled at baseline for BP/TC and patients on ≥ 7 regular medicines.

We also explored those changes in most economically deprived patients to assess the equity impact of the intervention.

### 2.14 Characterizing uncertainty

We used the nonparametric bootstrapping (Barber and Thompson, 2000) with 10,000 iterations to compute 95% confidence intervals (CIs) for incremental costs, incremental QALYs, ICERs, and ICURs. Bootstrapped ICERs and ICURs were plotted on the cost-effectiveness planes.

Despite effectiveness not being established, cost-effectiveness acceptability curves (CEACs) were experimented (van Hout et al., 1994; Fenwick et al., 2001; Fenwick et al., 2004; Fenwick et al., 2006), as this was a proof-of-concept study, to estimate the level of uncertainty associated with the decision regarding cost-effectiveness. The CEAC estimates the probability of the intervention being cost-effective over a range of values of the threshold cost per mmHg or per QALY.

We undertook a sensitivity analysis that seeks to assess the effect of changes in one key parameter (BP) on the results of the base-case analysis. In this average-case scenario, we use the average BP decrease derived from a meta-analysis of 39 randomized controlled trials that established the effectiveness of pharmacy-based hypertension management for intervention patients (Santschi et al., 2014), in intervention patients 6 months post-baseline. The pooled average systolic BP decrease (−7.6 mmHg) is similar to the results of a quasi-experimental controlled trial conducted in Portuguese pharmacies comprising 109 patients (−7.0 mmHg) (Costa, 2001).

We reported the revised point estimate and 95% CI from sensitivity analysis.

### 2.15 Engagement with patients and providers

We established a trial management group that held regular meetings with GPs, nurses, and community pharmacists (quality circles) before and during the trial. Feedback from providers contributed to identifying constraints in the trial and led to early minor adjustments in patient inclusion criteria and in intervention care pathways, which are reported in the separate effectiveness study.

Patients were not involved, but researchers engaged with intervention patients in a focus group to inform attributes and levels for a separate preference study.

## 3 Results

### 3.1 Participant flow

A total of 302 patients were invited, 214 accepted, and 203 (131 intervention and 72 control) patients entered the study and were included in the 6-month cost analysis. A total of 181 (116 intervention and 65 control) were included in the 6-month quality-of-life analysis.

**FIGURE 1:**
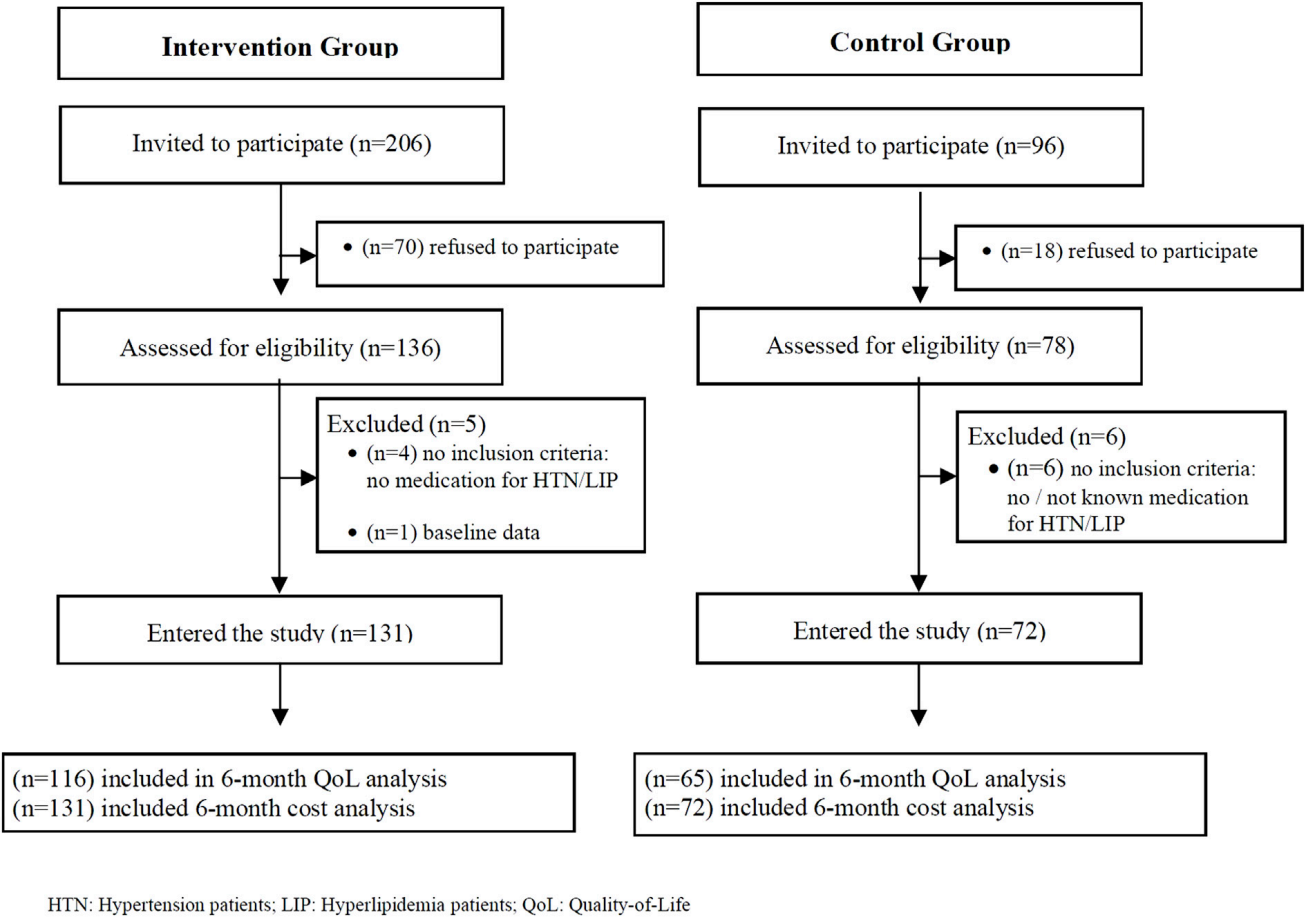
Flowchart of patients.

Figure 1 provides an overview of patient flow.

### 3.2 Baseline data

The mean age of patients was 64 (control) and 66 (intervention) years, most patients were female, 68% (control) and 74% (intervention) are either retired/pensioners, permanently disabled, unemployed, or performing household tasks, 8% (control) and 10% (intervention) are smokers, the mean number of comorbidities per patient is 2.4 (control) and 1.8 (intervention), the mean number of regular medicines per patient is 5 (control) and 4.4 (intervention), most patients are either on antihypertensive medication or antihypertensive and lipid-lowering medication, the mean number of years since the onset of antihypertensive medication is 6.3 (control) and 5.4 (intervention) years, and patients’ EQ-5D-3L™ baseline utility is 0.73 (control) and 0.79 (intervention).

As expected, all trial patients differ from the representative sample of the Portuguese population used in the valuation study of EQ-5D-3L™ in Portugal in various demographics. The representative sample used in the valuation study of EQ-5D-3L™ in Portugal has fewer females (53.3%), is considerably younger (mean age 50 ± 18.9), has a lower proportion of low-level educated individuals (38%), a lower proportion of retired/disabled/unemployed/on household task individuals (42.4%), and only 45.3% have a chronic disease (Ferreira et al., 2014).

The intervention and control patients are similar in gender distribution, employment status, most clinical variables, such as smoking status, number of comorbidities, number of regular medicines, number of years since the onset of medication, and number of antihypertensive medicines, and antihypertensive and lipid-lowering medication classes. Patients also have no significant difference in EQ-5D-3L™ baseline utility.

At baseline, intervention patients have significantly more compulsory education years, have a higher monthly equivalent income per person, are less below the at-risk poverty monthly threshold and have a lower BMI (*p* < 0.05). These variables were covariates in the analysis of QALYs.

Demographics, socioeconomic, and clinical characteristics for patients included at baseline are shown in Table 1.

**TABLE 1:** Patient demographics and clinical variables at baseline.


Demographics and case mix at baseline	Intervention (*n* = 116)	Control (*n* = 65)	*p*-value for difference^a^
Gender (C)			0.9438
Female, n (%)	72 (62.1%)	40 (61.5%)	
Male, n (%)	44 (37.9)	25 (38.5)	
Age, years (mean ± SD) (NR = 5 G2) (C)	65.8 (10.9)	64.0 (9.8)	0.0988
Education (NR = 12) (C)			
No. years compulsory education, mean (SD)	9.1 (4.6)	7.4 (4.6)	0.0214
Education ≤ elementary school 3rd cycle (current 9th grade/former 5th grade/technical schools), n (%)	64 (58.7)	45 (75.0)	0.0343
NR	7	5	
Employment status (C)			
Retired/pensioner + permanently disabled + unemployed + household tasks, n (%)	81 (74.3%)	41 (68.3%)	0.4065
NR	7	5	
Income status			
Approx. monthly equivalent income per person (=household income average threshold/no. of individuals in household) in € (SD) NR = 50	846.30 (569.14)	614.52 (411.54)	0.0058
Approx. household monthly income* (=household income average threshold) in € (SD) NR = 48 (C)	1,282.76 (854.84)	943.48 (602.83)	0.0204
≤ €501,20 (n, %)	23 (26.7%)	22 (48.9%)	0.0113
NR	30	20	
Municipality purchasing power index	95	92,5	
Smoking status (n, %) (C)			
Smoker (Y)	11 (9.9%)	5 (8.3%)	0.7355
NR	5	5	
BMI (mean kg/m^2^ ± SD) (C)	27.0 ± 4.2	28.4 ± 4.5	0.0426
Comorbidities** (NR = 5)			
No. comorbidities per patient (mean ± SD)	1.8 (1.6)	2.4 (1.8)	0.0506
≥1 (n, %)	87 (75.7)	56 (91.8)	0.0090
Ischemic heart disease: hypertension	24 (20.9)	25 (41.0)	0.0046
Gastroesophageal reflux disease	22 (19.1)	14 (23.0)	0.5499
Anxiety	27 (23.5)	13 (21.3)	0.7441
Depression	23 (20.0)	12 (19.7)	0.9586
Congestive heart failure	15 (13.0)	11 (18.0)	0.3747
No. regular medicines per patient*** (B)			
Mean, (SD)	4.4 (2.6)	5.0 (2.9)	0.2996
Minimum—Maximum	0–12	0–13	
Patients on (A, B, C)			
Antihypertensive medication (n, %)	37 (31.9%)	16 (24.6%)	0.0837
Lipid-lowering medication (n, %)	29 (25.0%)	10 (15.4%)	
Antihypertensive and lipid-lowering medication (n, %)	50 (43.1%)	39 (60.0%)	
Number of years since onset (mean ± SD) (C)			
Antihypertensive medication	5.4 (5.6)	6.3 (6.7)	0.8698
Lipid-lowering medication	4.4 (4.4)	5.9 (6.8)	0.4041
Antihypertensive medication (B)			
No. antihypertensive medicines per patient (mean ± SD)	1.5 (0.7)	1.7 (1.1)	0.4692
ACEI/ARB (C09)	75 (65.2%)	45 (73.8%)	0.2463
Alpha-blocker (C02CA)	1 (0.9%)	1 (1.6%)	1.0000
Beta-blocker (C07)	20 (17.4%)	15 (24.6%)	0.2548
Loop diuretics (C03CA + C03CA)	9 (7.8%)	8 (13.1%)	0.2584
Thiazides (C03A)	0 (0.0%)	0 (0.0%)	NA
Calcium channel blocker (C08)	9 (7.8%)	12 (19.7%)	0.0210
Other (C02 + C03)-(C02CA + C03A + C03CA + C03CB)	11 (9.6%)	6 (9.8%)	0.9538
Lipid-lowering medication (B)			
Statin (C10AA + C10BA + C10BX)	61 (53.0%)	35 (57.4%)	0.5827
Ezetimibe (C10AX09)	1 (0.9%)	1 (1.6%)	1.000
Fibrates (C10AB)	2 (1.7%)	2 (3.3%)	0.6101
Other (C10-(C10AA + C10BA + C10BX + C10AB + C10AX09)	0 (0.0%)	0 (0.0%)	NA
EQ-5D-3L™ baseline (t0) utility (mean, SD)	0.79 (0.22)	0.73 (0.22)	0.0793

BMI, body mass index; ACEI, angiotensin-converting enzyme inhibitor; ARB, angiotensin II receptor blockers; NR, nonrespondents; A, pharmacy dispensing software; B, primary care software; C, telephone baseline survey.

* Derived from monthly household income, as described.

** Derived from prescribed medicines using Rx-Risk Comorbidity Index as described. Top 5 comorbidities of all patients presented.

*** Number of medicines equals number of different INNs. (a) Wilcoxon–Mann-Whitney/Chi-square.

See Supplementary File S2 (Results: Quality-of-Life) for a detailed comparison between the proportion of representative sample of the Portuguese population, intervention, and control patients in severity level by EQ-5D dimension and EQ-VAS score.

At baseline, intervention and control patients had no significant difference in EQ-5D-3L™ baseline score (*p* = 0.0793), but intervention patients presented a higher EQ-VAS™ score (*p* = 0.0139).

At 6 months, intervention and control patients had no significant difference in EQ-5D-3L™ or EQ-VAS™ scores and there is no significant improvement in the mean change baseline/6 months.

Mean change baseline/6 months in EQ-5D-3L™ was 0.05 ± 0.23 for the intervention subgroup of uncontrolled patients (for BP/TC) at baseline and 0.07 ± 0.36 for the intervention subgroup of patients on ≥ 7 regular medicines, denoting a higher effect size, but this is not significant within nor between groups.

The mean change baseline/6 months in EQ-5D-3L™ was −0.12 ± 0.29 for the intervention subgroup of most income-deprived patients, but it is not significant either. However, the difference in mean change between intervention and control subgroups was significant (*p* = 0.0305) with the control subgroup reporting an improvement of 0.11 ± 0.24.

### 3.3 Resource use and costs

Estimated unit costs and sources are reported in Table 2. Costs were adjusted to 2018.

**TABLE 2:** Unit costs attached to different items of resource use.

Item	Estimated unit cost in 2018 (€)
GP visit^a^	37.07
Nurse visit^b^	16.16
Pharmacy visit—first (under ICP)^c^	22.50
Pharmacy visit—follow-up (under ICP)^c^	11.25
Lipid profile (TC + HDL + LDL + TG)	7.37
BP measurement^b^	2.52
TC test^b^	1.31
HDL test^b^	1.92
LDL test^b^	2.42
TG test^b^	1.72
Hospital outpatient visit^b^	34.44
Primary care ER visit^b^	36.36
Hospital ER visit^b^*	86.76
Hospital admission (cost per bed per day)^b^**	708.09
Rate per km (car)^d^	0.40
Patient time travel (cost per minute)^e^	0.1252

GP, general practitioner; TC, total cholesterol; HDL, high-density lipoprotein cholesterol; LDL, low-density lipoprotein cholesterol; TG, triglycerides; ER, emergency room.

a Gouveia et al.(2012).

b NHS Tariff GDH—Portaria n^º^ 207/2017, 2017.

c CEFAR (2018) estimate of pharmacy cost per minute based on published resource use and costs.

d Portaria n.^º^ 1553-D/2008, updated by Decreto-Lei n.^º^ 137/2010.

e Formula was adapted by authors from Mitchell and Bates, 2011 and detailed in the Methods section.

* Assumed midpoint ER classification of NHS tariff GDH—Portaria n^º^ 207/2017.

** Assumed most frequent cause of hospital admission in hypertension/hyperlipidemia—ischemic stroke GDH code 45, severity level 1.

Mean resource use item per patient considers patients who used that health care resource item. Mean cost item per patient considers all patients who reported resource use data for that cost item and may or may not have used that health care resource item.

In the 6 months prior to trial onset (baseline), intervention patients already had significantly higher costs in GP visits, lower costs for nurse visits, lower hypertension and/or lipid-lowering medication costs, lower travel and waiting time cost for nurse visits, and lower transportation costs to GP and nurse visits than control patients, but there is no difference in point-of-care measurement costs.

Patients reported no hospital admissions or working days lost due to hypertension/hyperlipidemia prior to the trial.

The mean total baseline costs per patient were €313.50 for intervention patients and €323.05 for control patients resulting in a mean cost difference of €9.55 per patient.

At 6 months after the onset of the trial, the highest trial cost was GP visits (42% of total intervention costs and 40% of control costs). The second most influential cost component was medication for hypertension and hyperlipidemia (24% of total intervention costs and 34% of control costs). The third highest cost was nurse visits (10% of total intervention costs and 11% of control costs). The cost of pharmacy visits ranks fourth (8% of total intervention costs). The fifth most influential cost component was patient travel and waiting time for GP visits (5% of total intervention costs and 6% of control costs).

The mean number of GP (4.17 ± 2.74) and nurse visits (4.0 ± 5.3) per patient over the 6-month trial in intervention patients denotes an increase from baseline. The mean number of nurse visits per patient is now higher than in the control group but not statistically significant. The mean number of pharmacy visits per patient in this period was lower at 1.75 ± 0.73.

There were no differences in the remaining cost items over the trial period between intervention and control patients, except for transportation costs to GP and nurse visits, which remained lower for intervention patients. However, USF point-of-care costs increased from baseline but not significantly.

Patients reported no hospital admission due to hypertension/hyperlipidemia over the trial.

Pharmacy-based intervention costs (pharmacy visits, pharmacy BP measurements, and pharmacy lipid profile tests) per patient were €37.89. If we add up patient travel and transportation costs to the pharmacy, this becomes €42.83 per patient.

The mean total trial costs per patient were €365.94 (intervention) and €325.15 (control), resulting in a mean cost difference of €40.79.

Trial costs between baseline and 6 months are reported in Table 3.

**TABLE 3:** Trial mean (SD) levels of resource use data with associated costs (€).

Cost item trial (T0+6 months)	G1 N	G1 total (n)	G1 mean (SD)	G2 N	G2 total (n)	G2 mean (SD)	*p*-value
GP visits cost	128	534 (128)	4.17 (2.74) 154.65 (101.40)	68	241 (68)	3.54 (2.40) 131.38 (88.85)	0.1383 0.1383
Nurse visits cost	128	287 (72)	4.0 (5.3) 36.23 (71.14)	68	149 (45)	3.3 (3.1) 35.41 (47.24)	0.2825 0.0890
Pharmacy visits cost	131	229 (131)	1.75 (0.73) 30.92 (8.18)	—	0 (0)	—	—
Medication HTN/LIP cost	112	1,010 (112)	9.02 (6.00) 87.21 (89.53)	66	717 (66)	10.86 (7.52) 111.16 (104.02)	0.1374 0.1023
Pharmacy BP Measurements cost	95	190 (95)	2.00 (0.80) 5.04 (2.01)	—	0 (0)	—	—
Pharmacy lipid profile (CT, LDL, HDL, TG) tests cost	87	128 (87)	1.47 (0.50) 1.93 (0.66)	—	0 (0)	—	—
USF BP measurements cost	93	216 (93)	2.32 (3.15) 5.85 (7.94)	58	86 (58)	1.48 (1.41) 3.74 (3.57)	0.0858 0.0858
NHS TC tests cost	86	60 (86)	0.70 (0.69) 0.91 (0.90)	50	24 (50)	0.48 (0.65) 0.62 (0.85)	0.0559 0.0559
NHS HDL tests cost	86	60 (86)	0.70 (0.69) 1.33 (1.31)	50	24 (50)	0.48 (0.65) 0.92 (1.23)	0.0559 0.0559
NHS LDL tests cost	86	57 (86)	0.66 (0.64) 1.60 (1.56)	50	24 (50)	0.48 (0.65) 1.16 (1.56)	0.0816 0.0816
NHS TG tests cost	86	59 (86)	0.69 (0.67) 1.18 (1.16)	50	24 (50)	0.48 (0.65) 0.83 (1.11)	0.0621 0.0621
Hospital outpatient visits cost	99	2 (2)	1.0 (0.0) 0.70 (4.87)	59	0 (0)	—	—
Primary care ER visits cost	99	0 (0)	—	59	2 (2)	1.0 (0.0) 1.23 (6.64)	—
Hospital ER visits cost	99	2 (2)	1.0 (0.0) 1.75 (12.27)	59	1 (1)	1.0 (−) 1.47 (11.30)	- 0.8911
Days in hospital cost	99	0 (0)	—	59	0 (0)	—	—
Travel + waiting time to GP visits cost	93	14,809 (93)	159.24 (139.09) 19.72 (17.44)	46	7,242 (46)	157.43 (214.33) 19.29 (26.70)	0.1610 0.1415
Travel + waiting time to nurse visits cost	93	7,110 (93)	76.45 (148.05) 8.09 (17.38)	46	3,293 (46)	71.59 (94.78) 7.63 (11.40)	0.3003 0.3125
Travel + waiting time to pharmacy visits cost	94	2,851 (94)	30.33 (22.75) 3.79 (2.85)	—	0 (0)	—	—
Transportation costs for GP visits	85	265.31 (85)	3.12 (7.11)	44	228.55 (44)	5.19 (6.44)	0.0042
Transportation costs for nurse visits	85	65.38 (85)	0.77 (1.70	44	225.20 (44)	5.12 (15.92)	0.0028
Transportation costs for pharmacy visits	96	110.01 (96)	1.15 (3.83)	—	0 (0)	—	—
Total			365.94			325.15	

G1, intervention; G2: control; GP, general practitioner; HTN/LIP, hypertension/lipid-lowering; USF, primary care family health unit; BP, blood pressure; NHS, national health service; TC, total cholesterol; HDL, high-density lipoprotein cholesterol; LDL, low-density lipoprotein cholesterol; TG, triglycerides; ER, emergency room.

### 3.4 Cost-utility

#### 3.4.1 Incremental costs and QALYs

The mean QALY for the 6-month trial using the AUC was 0.4040 ± 0.0855 for the intervention group and 0.3855 ± 0.1093 for the control group, resulting in a decremental (after adjustment) QALY of −0.0042, albeit not significant (95% CI -0.0253; 0.0170). The mean incremental cost per patient over 6 months (vs. control) was €40.79 (95% CI −18.10; 94.92), and we estimated the ICUR to be €9,711.99 (95% CI −56,968.18; 55,293.82) per each decremental utility in QALY albeit uncertainty reflected in the 95% CI (Table 4).

**TABLE 4:** Incremental QALY and incremental costs.

QALY	Mean (SD)	CI (lower)	CI (upper)
Intervention (*n* = 89)	0.4040 (0.0855)	0.3860	0.4220
Control (*n* = 46)	0.3855 (0.1093)	0.3530	0.4180
Incremental^a^ (unadjusted)	0.0185	−0.0164	0.0539
Incremental^a^ (adjusted*)	−0.0042	−0.0253	0.0170

Costs	Mean	CI (lower)	CI (upper)
Intervention	365.94	330.71	400.357
Control	325.15	280.31	372.52
Incremental^a^	40.79	−18.10	94.92
ICUR	−9,711.99	−56968.18	55,293.82

* Adjusted for EQ-5D baseline utility and patient baseline characteristics.

a Non-parametric confidence intervals based on 10,000 bootstrap replicates for incremental costs, QALYs, and ICUR.

#### 3.4.2 Cost-effectiveness plane

**FIGURE 2:**
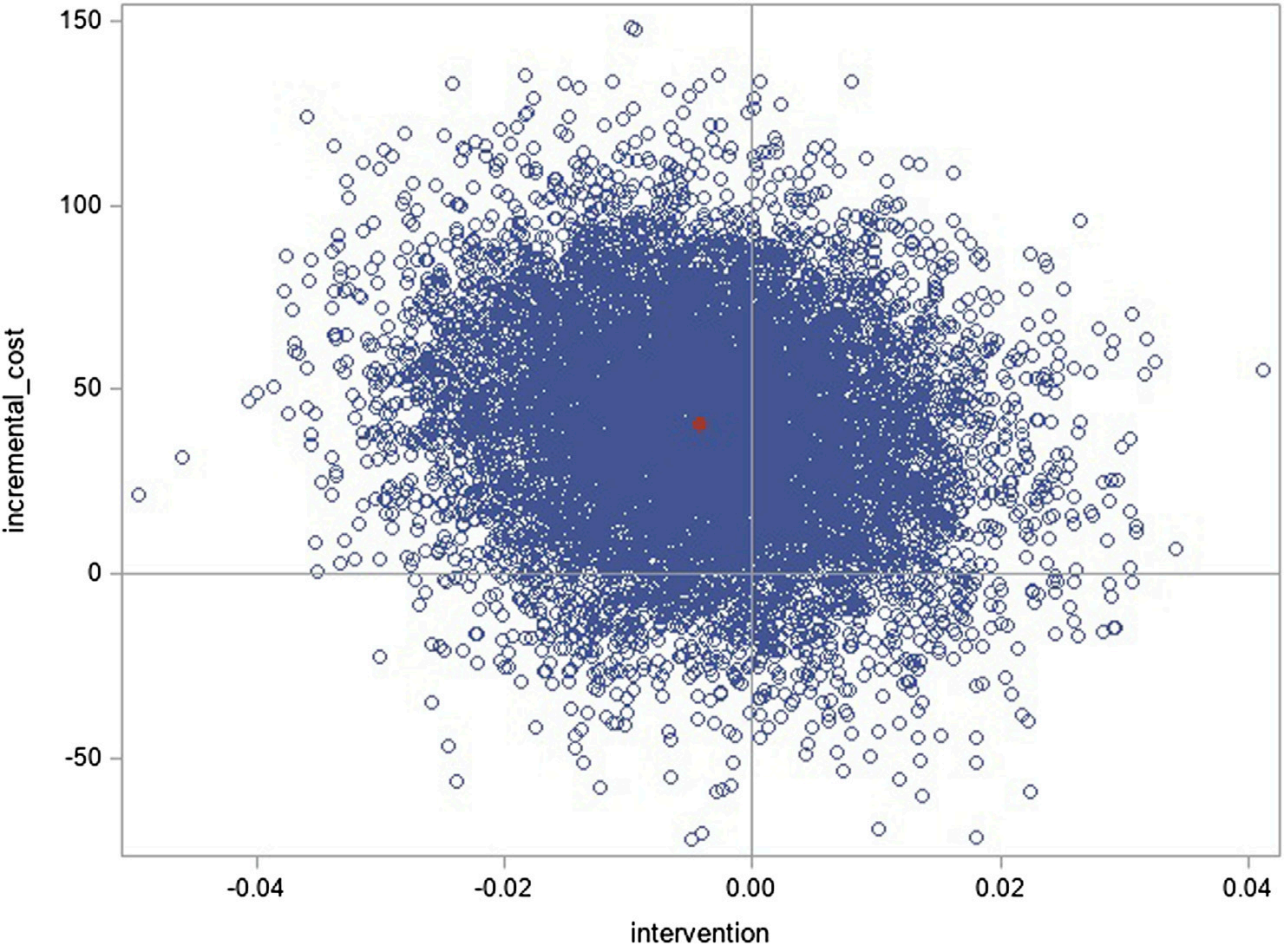
Cost-effectiveness plane (ICUR).

The bootstrapped ICURs on the cost-effectiveness plane show predominance in the northwest quadrant (less effective and more costly); hence, this intervention is dominated despite some uncertainty falling in the northeast quadrant (Figure 2).

#### 3.4.3 Cost-effectiveness acceptability curve

**FIGURE 3:**
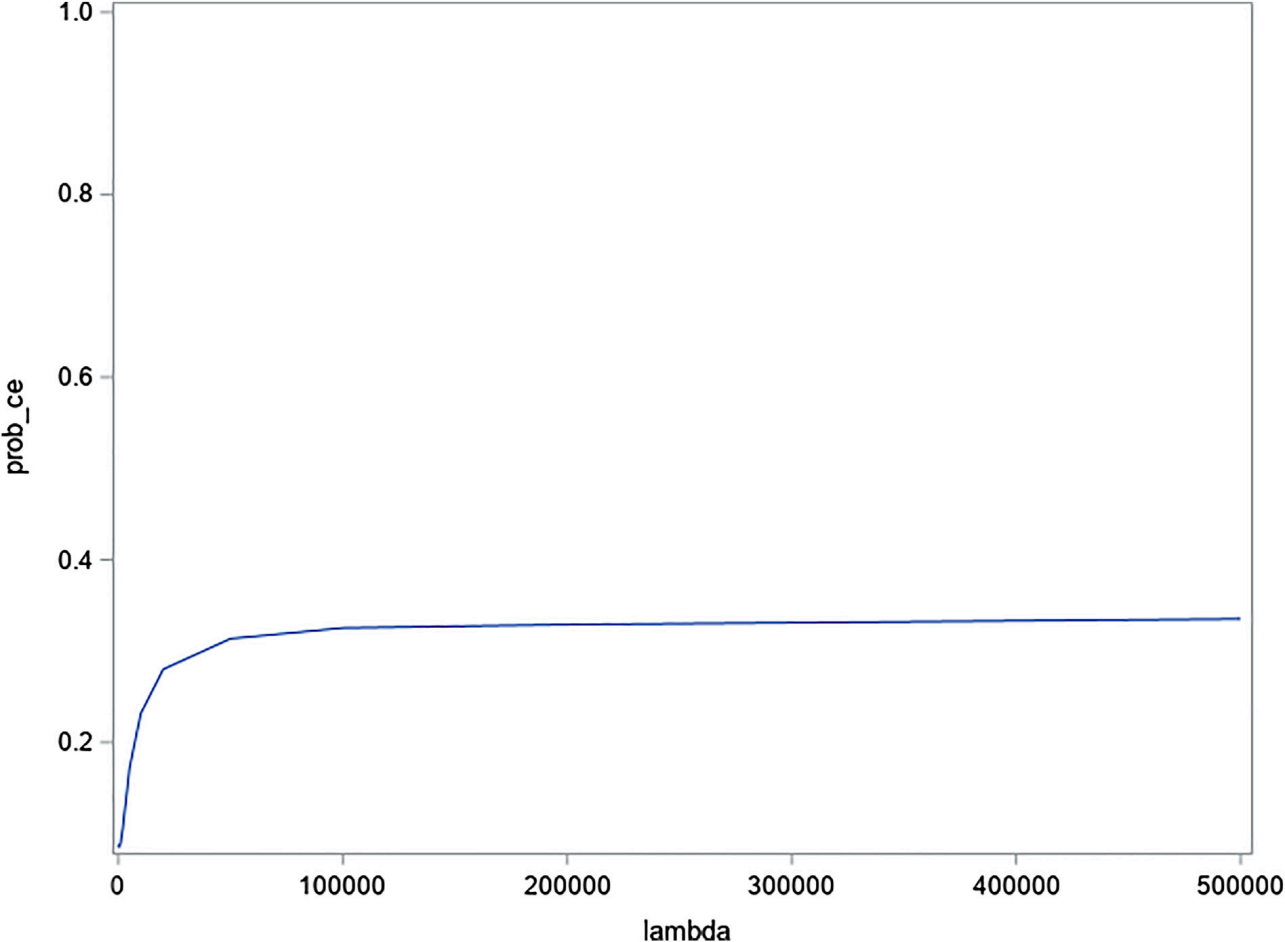
CEAC (Qaly).

The probability of the intervention being cost-effective at the €20,000 per QALY threshold value vs. control is 28% only and remains below 40% at higher threshold values (Figure 3).

### 3.5 Cost-effectiveness

As previously stated, we used the results of the effectiveness study (blood pressure change) to perform a proof-of-concept cost-effectiveness analysis despite no significant change observed for blood pressure in the effectiveness study.

#### 3.5.1 Incremental costs and effectiveness

The mean incremental cost per patient over 6 months (vs. control) was €10.33 (95% CI –62.64; 82.03), and we estimated the ICER at −€4.01 (95% CI –31.85; 30.95) per each 1 mmHg systolic BP decrease and at €3.60 (95% CI –53.94; 51.72) per mmHg diastolic BP increase. We can also observe the uncertainty expressed in the wide confidence intervals (Table 5).

**TABLE 5:** Incremental effectiveness (BP).

Effectiveness (BP)	Mean	CI (lower)	CI (upper)
Incremental effectiveness SBP	−2.57	−29.55	24.41
Incremental effectiveness DBP	2.87	−12.0	17.74

Costs (BP)	Mean	CI (lower)	CI (upper)
Incremental costs^a^	10.33	−62.64	82.03
ICER SBP^a^	−4.01	−31.85	30.95
ICER DBP^a^	3.60	−53.94	51.72

BP, blood pressure; SBP, systolic blood pressure; DBP, diastolic blood pressure.

a Non-parametric confidence intervals based on 10,000 bootstrap replicates for incremental effectiveness.

#### 3.5.2 Cost-effectiveness planes

**FIGURE 4:**
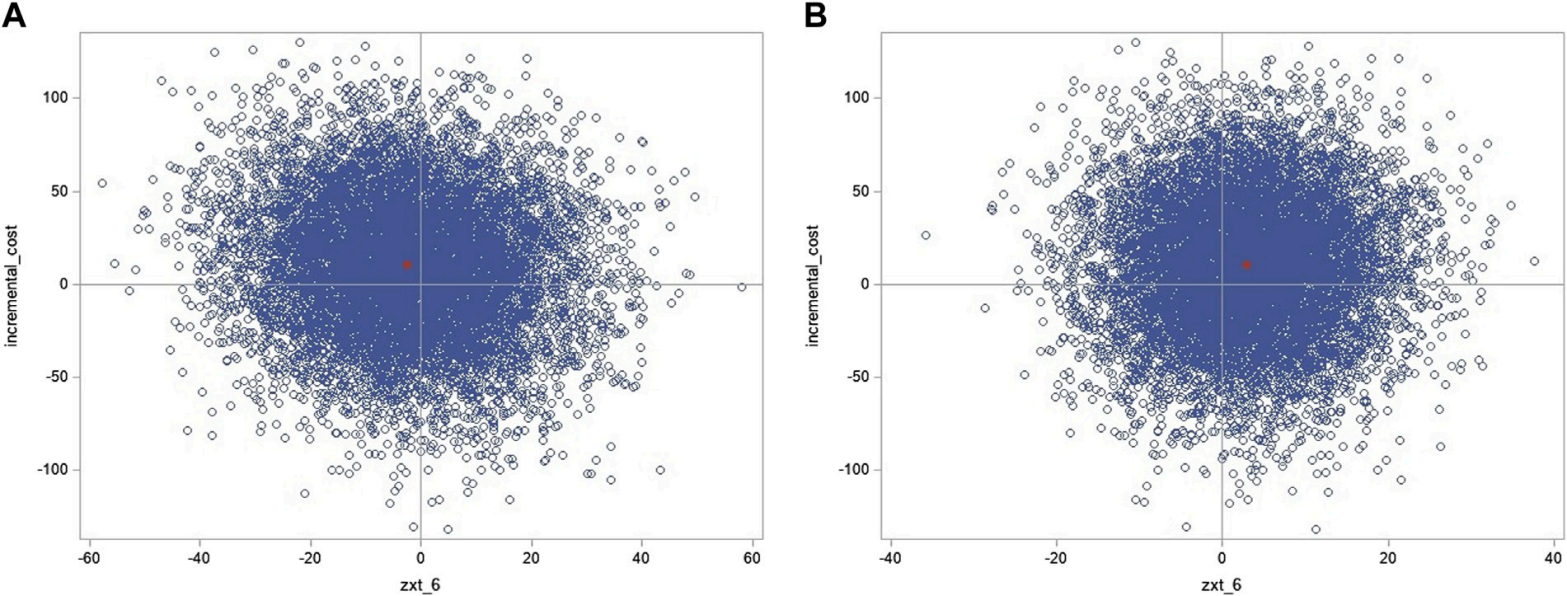
Cost-effectiveness plane base-case (ICER for **(A)** systolic and **(B)** diastolic blood pressure).

The bootstrapped ICERs on the cost-effectiveness planes show slight predominance in the northwest quadrant for systolic BP, which, in this case, means more costly and more effective, but more costly and less effective when considering diastolic BP. However, there is considerable uncertainty (Figure 4).

#### 3.5.3 Cost-effectiveness acceptability curves

**FIGURE 5:**
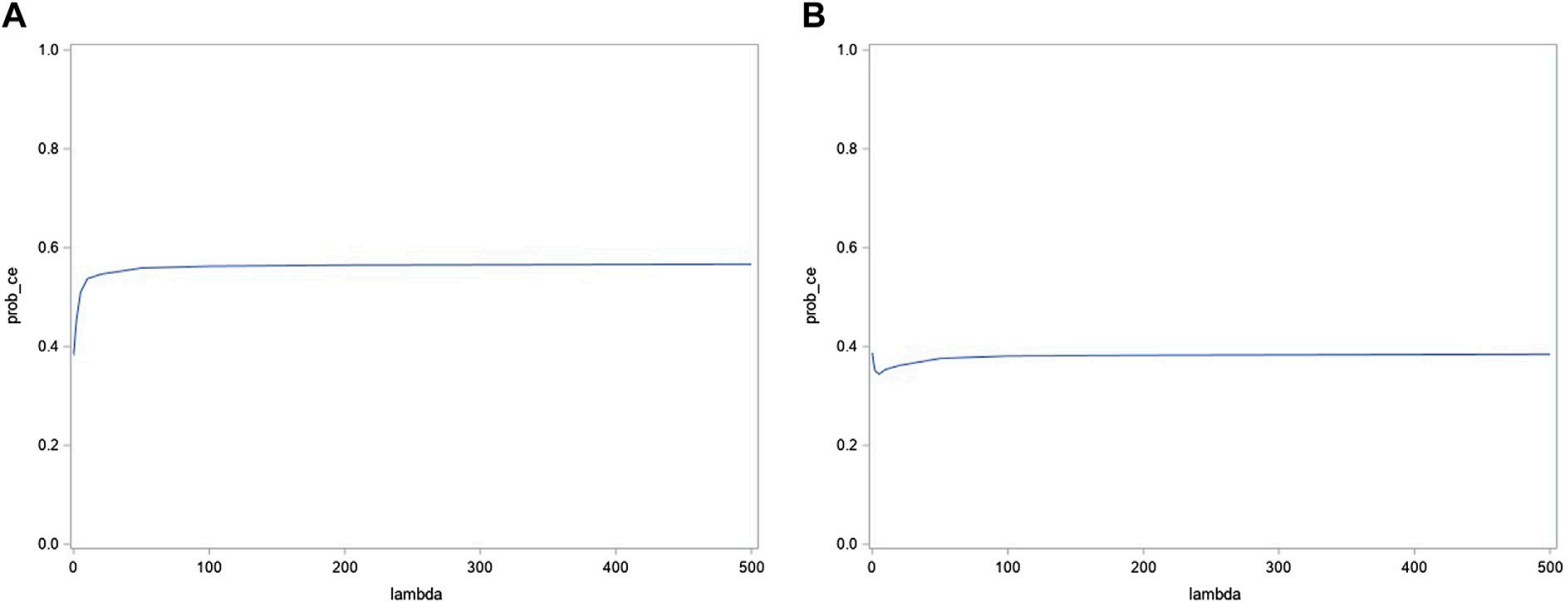
CEAC base-case **(A)** systolic and **(B)** diastolic blood pressure.

The probability of this intervention being cost-effective at the ICER threshold of €500 per mmHg decrease vs. control is 57% for systolic BP and 38.5% for diastolic BP (Figure 5). We report this threshold as there is no substantial change in the probability of the intervention being cost-effective even at the highest ICER threshold depicted (€500 mmHg).

### 3.6 Sensitivity analysis (cost-effectiveness)

The ICER, associated 95% CI, and CEAC values were estimated for an “average case” scenario when applying the mean change in systolic BP of –7.6 mmHg (95% CI –9.0; –6.3) and mean change in diastolic BP of –3.9 mmHg (95% CI –5.1; –2.8) derived from the meta-analysis (Santschi et al., 2014), all else being equal. The mean incremental cost is €36.82 (95% CI –25.03; 99.51). The revised ICER was now estimated to be €4.80 (95% CI –14.70; 13.90) per each 1 mmHg systolic BP decrease and €9.40 (95% CI –21.70; 21.30) per each 1 mmHg diastolic BP decrease.

**FIGURE 6:**
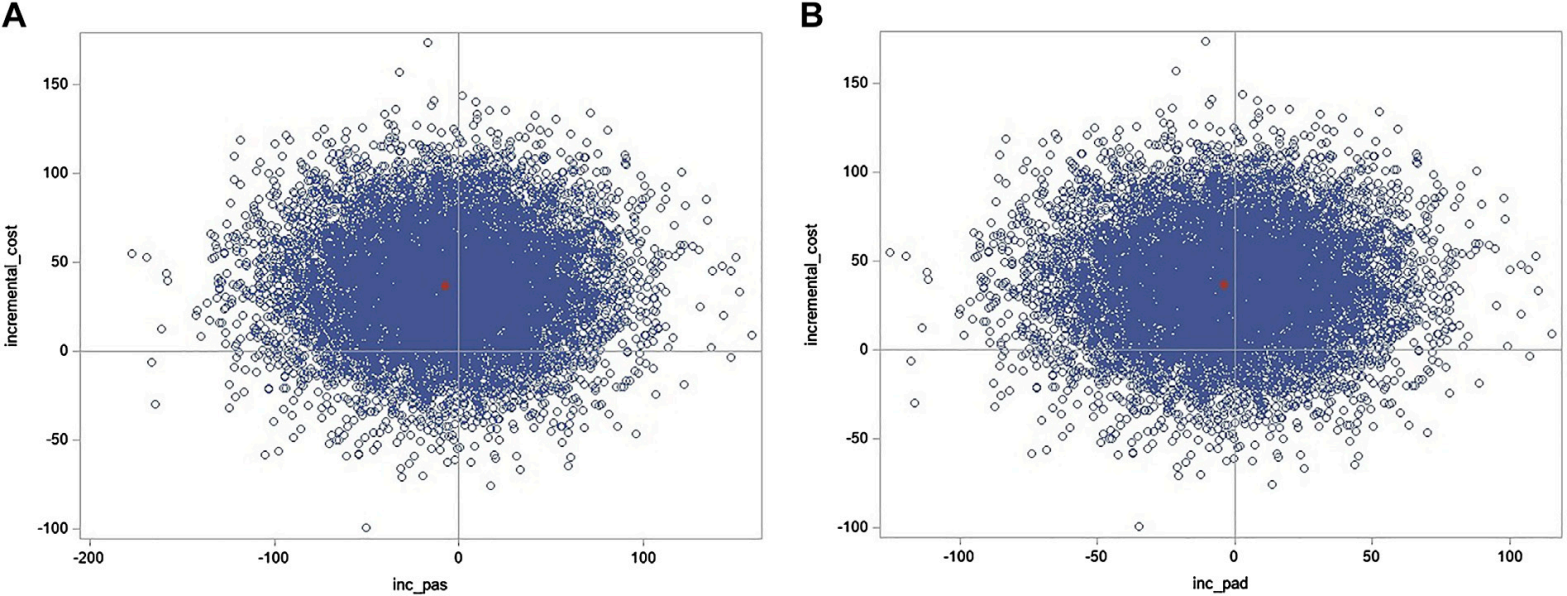
Cost-effectiveness plane average-case (ICER for **(A)** systolic and **(B)** diastolic blood pressure).

The revised bootstrapped ICERs on the cost-effectiveness planes now demonstrate a slight shift upwards and left to the northwest quadrant, which would mean a more costly and, in this case, more effective (BP decrease) intervention despite some uncertainty, which still falls in the northeast quadrant (Figure 6).

**FIGURE 7:**
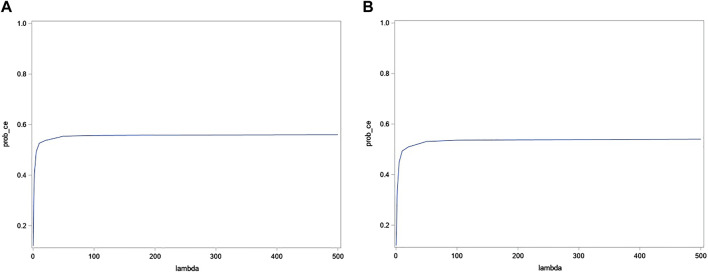
CEAC average-case **(A)** systolic and **(B)** diastolic blood pressure.

The probability of this intervention being cost-effective at the ICER threshold of €500 per mmHg decrease vs. control using effectiveness data from the literature is 56% for systolic BP and 54% for diastolic BP. The revised probability for systolic BP is not that different from the base case (Figure 7).

## 4 Discussion

### 4.1 Key findings

This was a proof-of-concept study to experiment and explore both methods and results of the first CEA and CUA in Portugal alongside a pragmatic controlled trial of a pharmacy collaborative health intervention with primary care using interprofessional communication technology-driven under real-world conditions in Portugal.

The intervention has not shown cost-effectiveness or cost-utility when compared to usual care as denoted by a high level of uncertainty expressed in wide confidence intervals. The probability of the intervention being cost-effective at the threshold of €20,000 per QALY gained is 28% and 57% at the threshold of €500 per mmHg systolic BP decrease.

### 4.2 Limitations

We could not achieve a large enough sample size to rule out uncertainty as expressed by the wide confidence intervals and lack of significance in the effect which affected cost-effectiveness. The low sample size was due to difficulty in recruitment, which was likely the consequence of the preceding events, as further outlined.

Two major trial limitations have negatively affected effectiveness and costs, hence, cost-effectiveness: partial failure of data exchange between providers directly affected the effectiveness and, as a consequence, no delegation of projected tasks from USF to pharmacies, which, in turn, increased trial costs.

Although we successfully implemented and tested IT developments for data exchange between providers in the initial Vitacare^®^ clinical software, its private proprietor unexpectedly pleaded insolvency as we were about to start the trial. This was replaced by the public clinical software SClínico^®^, but most IT developments and interoperability were not possible or became available extremely late in the new primary care software.

As a consequence of partial failure to communicate pharmacy decisions to GPs, partial transfer of tasks from GPs and nurses to pharmacies did not occur. The number of GP and nurse visits and USF point-of-care measurements in intervention patients increased (instead of decreasing) and the number of pharmacy visits decreased (instead of increasing), causing inefficiency. In addition, patient GP visits do not occur on the same date as nurse visits which duplicates patient travels to primary care causing additional inefficiency. This increased costs; hence, cost-effectiveness was not established either.

Other limitations fall on methods that can serve as lessons to improve in future trials.

EQ-5D-3L™ is not a sensitive instrument for capturing changes in quality of life in asymptomatic chronic diseases. Although we could have added a disease-specific instrument, for example, MINICHAL (Badia et al., 2002; Cunha, 2014), we did not use it to avoid an extra burden on the patient telephone surveys already containing a large data set collection. We did not use EQ-5D-5L™ as the new Portuguese value set was not available then (Ferreira et al., 2019).

Missing data in cost items resulted in different sample populations across items. Although we could have used multiple imputations, we used available-case analysis due to time constraints, but this precluded adjusting for baseline cost differences. Mean cost item per patient considering just patients who reported resource use data (versus all arm patients) led to a slight overestimation of costs, although the impact on the incremental cost was minimal.

Likely, the 6-month follow-up period of this study was not long enough to detect health-related resource use triggered by hypertension or hyperlipidemia.

Patients also reported health care resource use, so the quality of these data relied on patient recall, instead of medical records, and may have caused recall bias.

We did not collect data on the alternative use of time lost for the fraction aged above retirement age, which may have overestimated productivity costs for unpaid work. We believe the impact is likely not relevant as productivity costs represented less than 6% of costs.

Due to effectiveness not being established, we did not project beyond trial nor used a model.

We did not consider a best-case scenario in our sensitivity analysis for a partial transfer of GP and nurse visits to pharmacy visits.

### 4.3 Strengths

We sought to apply the classic non-welfare health economic evaluation techniques and methods for medicines to a public health collaborative intervention alongside a trial in Portugal based on previous work (Costa et al., 2019).

We used a societal perspective to capture all relevant costs and effects, including productivity costs.

We used patient-level resource use, costs, and outcomes collected alongside a pragmatic trial using different real-world data sources at several time points.

Change in quality of life is considered important for patient subgroups, including the economically deprived, in an attempt to capture equity considerations.

We have identified and detailed unit costs from relevant resources and further adjusted them to present value, which will be useful to guide future costing and economic evaluations of pharmacy-based interventions.

The sensitivity analysis used an average-case scenario based on the pooled average change of BP from a meta-analysis (Santschi et al., 2014). Furthermore, the change in systolic BP in this meta-analysis is consistent with the change obtained in a quasi-experimental controlled trial conducted in Portuguese pharmacies in 1999 (Costa, 2001).

### 4.4 Generalizability

Considering the trial limitations, the vast amount of evidence establishing effectiveness (Santschi et al., 2014), and some evidence establishing cost-effectiveness and cost-utility (Shireman and Svarstad, 2016; Elliott et al., 2017; Marra et al., 2017; Bosmans et al., 2019; Twigg et al., 2019) for pharmacy-based hypertension and hyperlipidemia management, our study findings are not generalizable for community pharmacy and primary care in Portugal.

## 5 Conclusion

### 5.1 Implications for policy and practice

It is essential to plan interventions for high-risk, uncontrolled, or economically deprived populations where pharmacists can add the most value.

Pre-agreed ICPs with the partial transfer of primary care tasks to pharmacies that work is also a key to preventing unnecessary duplication of tasks and driving value-based health care.

Policymakers should also address combined risk-share bundled payment models between pharmacies and primary care aligned with shared quality targets and stakeholder engagement.

Our findings offer lessons that can be applied in future economic evaluations of pharmacy collaborative interventions with the potential for reimbursement in various jurisdictions.

### 5.2 Implications for research

Ensuring well-designed ICPs can apply IT-driven communication between providers is crucial to driving effectiveness and cost-effectiveness.

Resource items and unit costs per resource item determined in this study may be useful to guide future TDABC costing and economic evaluations of pharmacy-based interventions.

The cost should be the expected fee for the service to be paid to the pharmacy, as would be the case if reimbursed by the payer, and similarly to the economic evaluation of medicines.

Future research could combine trial data with projected data beyond trial and a validated model since many benefits of public health interventions are often delivered well into the future yet using a reliable time horizon to enable linking intermediate to long-term outcomes.

Finally, this research may also contribute to exploring economic evaluation tools and methods in more complex public health interventions in general, aiming to shed light on the real-world impact of public health interventions and programs.

## Data Availability

The datasets presented in this article are not readily available because the raw data supporting the conclusions of this article will be made available by the authors on request after the last PhD paper of the first author is published. This includes anonymized, non-identifiable patient-level data linked to pharmacy and patient surveys in compliance with privacy regulations. Patient-level data linked to primary care was obtained from SPMS. Requests to access the datasets should be directed to the first author for patient-level data linked to pharmacy and patient surveys; https://www.spms.min-saude.pt/ for patient-level data linked to primary care.

## Glossary

ACEI: angiotensin-converting enzyme inhibitor
ACeS: Group of Primary Care Units [Agrupamento dos Centros de Saúde]
ACSC: Ambulatory Care Sensitive Condition
ADE: adverse drug event
ANF: National Association of Pharmacies [Associação Nacional das Farmácias]
ARB: angiotensin II receptor blocker
ARS: Regional Health Administration [Administração Regional de Saúde]
ATC: Anatomical Therapeutic Chemical
AUC: area under the curve
BMI: body mass index
BP: blood pressure
CBA: cost-benefit analysis
CEA: cost-effectiveness analysis
CEAC: cost-effectiveness acceptability curve
CEFAR: Centre for Health Evaluation and Research
CHEERS: Consolidated Health Economic Evaluation Reporting Standards
CI: confidence interval
CNP: national product code [Código Nacional de Produto]
CNPEM: National Code for Electronic Prescribing [Código Nacional de Prescrição Electrónica de Medicamentos]
CUA: cost-utility analysis
EQ-5D: EuroQol-5D (5 dimensions)
ER: emergency room
G1: intervention group
G2: control group
GDH: diagnostic-related groups [Grupos de Diagnóstico Homogéneos]
GDPR: General Data Protection Regulation
GLM: generalized linear model
GP: general practitioner
HDL: high-density lipoprotein cholesterol
HTN: hypertension
ICER: incremental cost-effectiveness ratio
ICHOM: International Consortium for Health Outcomes Measurement
ICP: integrated care pathway
ICUR: incremental cost-utility ratio
INE: National Institute of Statistics [Instituto Nacional de Estatística]
INN: international nonproprietary name
IPCC: Municipality Power Purchasing Index [Índice de Poder de Compra Concelhio]
IT: information technology
ITT: intention-to-treat
LDL: low-density lipoprotein cholesterol
LIP: hyperlipidemia
MAR: missing-at-random
NHS: National Health Service
OECD: Organization for Economic Co-Operation and Development
PIA: privacy impact assessment
QALY: quality-adjusted life year
QoL: quality-of-life
SPMS: Shared Services of Ministry of Health [Serviços Partilhados do Ministério da Saúde]
TC: total cholesterol
TDABC: time-driven activity-based costing
TG: triglycerides
USF: Primary Care Family Health Unit [Unidade de Saúde Familiar]
VAS: Visual Analog Scale
WHO: World Health Organization.
