# Supplementary material for: Cost-effectiveness and cost-utility of hypertension and hyperlipidemia collaborative management between pharmacies and primary care in portugal alongside a trial compared with usual care (USFarmácia®)
Source: Front Pharmacol. 2022 Sep 8;13:903270. doi: 10.3389/fphar.2022.903270 (PMC9493118; doi:10.3389/fphar.2022.903270)
Supplement: Supplementary file 1 [file DataSheet2.PDF]

Supplementary File 2

Results: Quality-of-Life

The proportion of all trial patients reporting EQ-5D-3L™ baseline severity for self-care, usual activities, and pain/discomfort does not seem to be different from the representative sample of the Portuguese population used in the valuation study of EQ-5D-3L™ in Portugal (Ferreira et al., 2014). In contrast, we seem to have more trial patients reporting EQ-5D-3L™ baseline severity for the mobility dimension. The proportion of control patients reporting baseline severity for pain/discomfort and anxiety/depression seems higher.

At 6 months, there seem to be fewer all trial patients reporting severity in almost all dimensions.

Intervention patients and the representative sample of the Portuguese population used in the valuation study of EQ-5D-3L™ in Portugal are similar in mean EQ-VAS™ baseline scores.

Comparison between the proportion of representative sample of Portuguese population, intervention and control patients in severity level by EQ-5D dimension and in EQ-VAS score is reported in **Supplementary Table 1** and **Supplementary Figure 1**.

SUPPLEMENTARY TABLE 1 | Proportion of patients reporting EQ-5D-3L severity level 2+3.

	**Baseline** (level 2 or 3)				**6 Months** (level 2 or 3)		
**EQ-5D-3L™**	**National**[a] **(n=450)**	**Intervention (n=116)**	**Control (n=65)**	NR	**Intervention (n=116)**	**Control (n=65)**	NR
Mobility (%)	16.7	21.2	32.1	24	15.8	26.3	23
Self-care (%)	4.8	5.8	5.7	24	5.0	8.5	21
Usual activities (%)	16.3	11.5	17.0	24	8.9	6.9	22
Pain / Discomfort (%)	44.7	40.4	48.1	23	31.0	36.2	23
Anxiety/ Depression (%)	34.4	26.0	40.7	23	18.8	17.2	22
**EQ-VAS** (mean ± SD)	76.9 (18.6)	76.4 (18.3)	68.6 (20.0)	23	77.6 (14.6)	71.4 (21.4)	27

NR: Nonrespondents.
[a]Ferreira et al., 2014

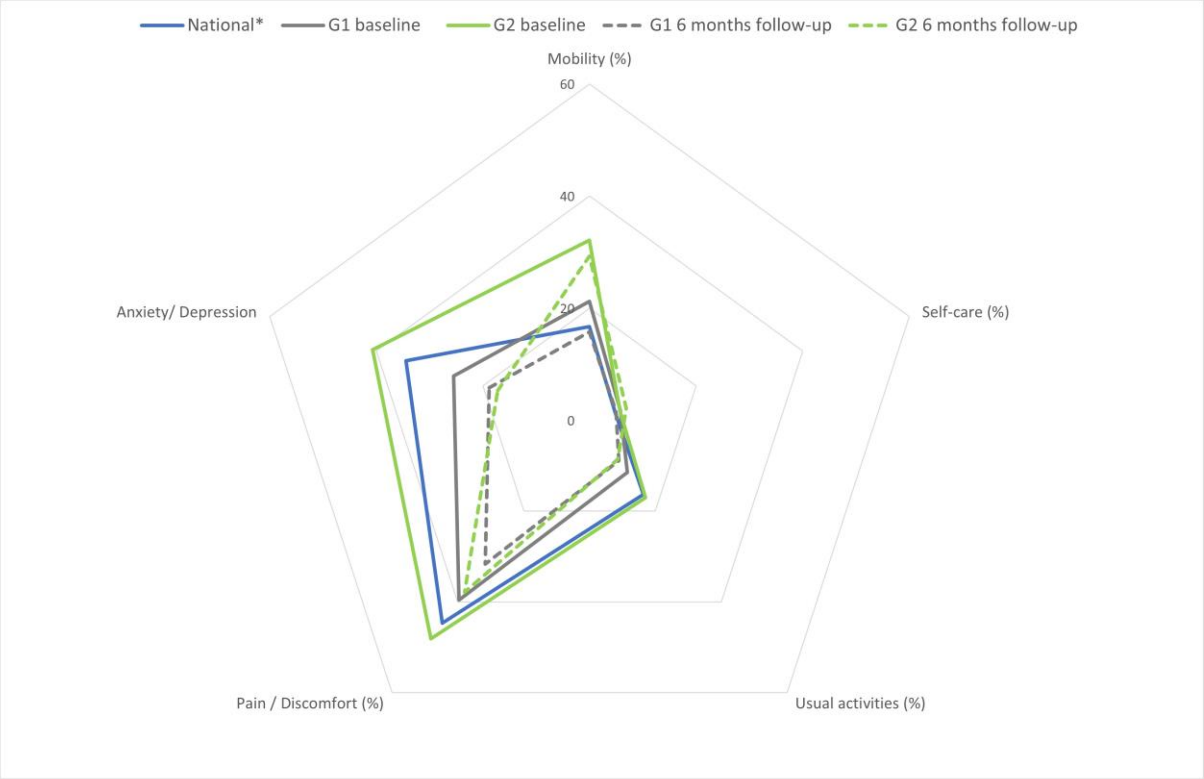

SUPPLEMENTARY FIGURE 1 | Proportion of patients reporting health problems (severity level 2/3) by EQ-5D-3L™ dimension.

References:

Ferreira, L.N., Ferreira, P.L., Pereira, L.N., Oppe, M. (2014). The valuation of the EQ-5D in Portugal. *Qual Life Res*. 23:2, 413-23. doi: 10.1007/s11136-013-0448-z .
