# Supplementary material for: Cost-effectiveness and cost-utility of hypertension and hyperlipidemia collaborative management between pharmacies and primary care in portugal alongside a trial compared with usual care (USFarmácia®)
Source: Front Pharmacol. 2022 Sep 8;13:903270. doi: 10.3389/fphar.2022.903270 (PMC9493118; doi:10.3389/fphar.2022.903270)
Supplement: Supplementary file 2 [file DataSheet1.PDF]

Supplementary File 1

Methods: Measurement of Patient Demographics, Socioeconomic, and Clinical Data

Other patient variables included:

- Socio-demographic variables: gender, age, education level, employment status, income status, Municipality Purchasing Power Index ('Índice de Poder de Compra Concelhio', or IPCC).

- Baseline clinical and treatment variables: smoking status, Body Mass Index (BMI), comorbidities, medication profile.

Income status presented included:

1) Monthly equivalent income per person calculated from the net monthly household income divided by the number of equivalent adults using the Organization for Economic Co-operation and Development (OECD)-modified scale which assigns a value of 1 to the first household adult, a value of 0.5 to each additional individual aged ≥14, and 0.3 to each child <14, and then adds these up (Eurostat, 2020). However, as we did not have information on the age of household members and due to the average age of our study population, we used 0.5 for each additional individual in the household with ≥ 2 members.

2) Patients below at-risk poverty monthly threshold < €501,20 (INE, PORDATA, 2018).

The medication profile presented included anti-hypertensive and/or lipid-lowering medications which were classified according to medication classes defined in the ICHOM Standard Set for Hypertension (International Consortium for Health Outcomes Measurement, 2017).

Comorbidities presented were defined using the Rx-Risk Comorbidity Index which lists 43 comorbidity categories, each mapped to a set of prescribed medicines according to the World Health Organization (WHO) ATC codes, providing an indirect method to describe patients' comorbidities based on prescribed medicines in the absence of medical diagnosis data (Pratt et al., 2018).

References:

Eurostat. Equivalised Disposable Income (2020). https://ec.europa.eu/eurostat/statistics-explained/index.php?title=Glossary:Equivalised_disposable_income [Accessed May 20, 2020].

INE, PORDATA (2018). Limiar de risco de pobreza 2018 (Poverty threshold 2018 in Portugal). https://www.pordata.pt/Portugal/Limiar+de+risco+de+pobreza-2167 [Accessed May 20, 2020].

International Consortium for Health Outcomes Measurement (2017). ICHOM Standard Set for Hypertension in Low and Middle Income Countries. https://www.ichom.org/portfolio/hypertension-in-low-and-middle-income-countries/ [Accessed May 20, 2020].

Pratt, N.L., Kerr, M., Barratt, J.D., Kemp-Casey, A., Kalisch Ellett, L.M., Ramsay, E., et al. (2018). The validity of the Rx-Risk Comorbidity Index using medicines mapped to the Anatomical Therapeutic Chemical (ATC) Classification System. *BMJ Open*. 8:4, e021122. doi: 10.1136/bmjopen-2017-021122. Erratum in: BMJ Open. 2020 Sep 6;10(9):e021122corr1
